# Resonant Tip-Enhanced Raman Spectroscopy of a Single-Molecule Kondo System

**DOI:** 10.1021/acsnano.4c02105

**Published:** 2024-05-07

**Authors:** Rodrigo Cezar de Campos Ferreira, Amandeep Sagwal, Jiří Doležal, Sofia Canola, Pablo Merino, Tomáš Neuman, Martin Švec

**Affiliations:** † Institute of Physics, Czech Academy of Sciences; Cukrovarnická 10/112, Praha 6 CZ16200, Czech Republic; ‡ Faculty of Mathematics and Physics, Charles University; Ke Karlovu 3, Praha 2 CZ12116. Czech Republic; § Institute of Physics, École Polytechnique Fédérale de Lausanne, Lausanne CH-1015, Switzerland; ∥ Instituto de Ciencia de Materiales de Madrid; CSIC, Sor Juana Inés de la Cruz 3, Madrid E28049, Spain; ⊥ Institute of Organic Chemistry and Biochemistry, Czech Academy of Sciences; Flemingovo náměstí 542/2. Praha 6 CZ16000, Czech Republic

**Keywords:** TERS, resonant Raman, PTCDA, Kondo, SPM, break-junction

## Abstract

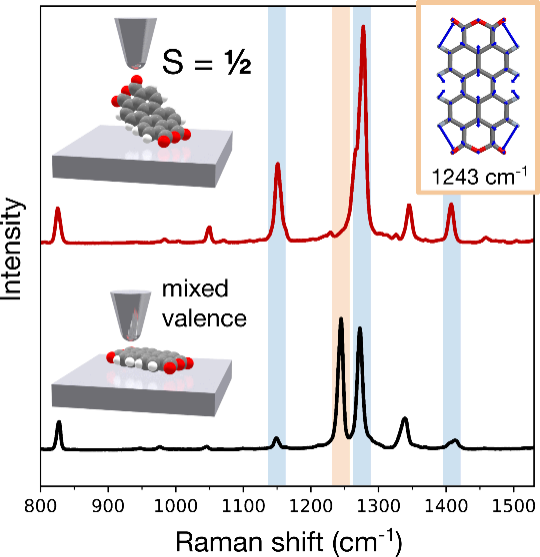

Tip-enhanced Raman spectroscopy (TERS) under ultrahigh vacuum and cryogenic conditions enables exploration of the relations between the adsorption geometry, electronic state, and vibrational fingerprints of individual molecules. TERS capability of reflecting spin states in open-shell molecular configurations is yet unexplored. Here, we use the tip of a scanning probe microscope to lift a perylene-3,4,9,10-tetracarboxylic dianhydride (PTCDA) molecule from a metal surface to bring it into an open-shell spin one-half anionic state. We reveal a correlation between the appearance of a Kondo resonance in differential conductance spectroscopy and concurrent characteristic changes captured by the TERS measurements. Through a detailed investigation of various adsorbed and tip-contacted PTCDA scenarios, we infer that the Raman scattering on suspended PTCDA is resonant with a higher excited state. Theoretical simulation of the vibrational spectra enables a precise assignment of the individual TERS peaks to high-symmetry A_g_ modes, including the fingerprints of the observed spin state. These findings highlight the potential of TERS in capturing complex interactions between charge, spin, and photophysical properties in nanoscale molecular systems and suggest a pathway for designing single-molecule spin-optical devices.

Precise local measurements and identification of individual single-molecule configurations based on their vibrational state fingerprints are a long-standing challenge in spectroscopy. Tip-enhanced Raman spectroscopy (TERS) operated in ultrahigh vacuum (UHV) has recently witnessed rapid advances in this direction.^1−3^ This technique employs a plasmonic nanocavity formed between a sharp tip apex and the substrate to enable bidirectional coupling between the electromagnetic far-field and near-field confined into subnanometric volumes, achieving submolecular spatial resolution.^4,5^ The TERS, along with other near-field techniques such as tip-enhanced photoluminescence (TEPL)^6−8^ and electroluminescence (EL),^9−13^ was integrated with the cryogenic scanning tunneling microscopy (STM) and permitted to optically probe single organic molecules adsorbed on atomically flat surfaces. TERS has been applied, e.g., for intramolecular mapping of vibrational modes in phthalocyanine and porphyrinoid molecules^2,3,14−16^ and tracking conformational changes, tautomerization, or deprotonation of polyaromatic hydrocarbons, using specific vibrational fingerprints.^17−19^ This technique has demonstrated the ability to provide precise optical fingerprints of individual functional molecules in various chemical, electronic, and charge states. Therefore, application of TERS is a possible pathway to a fast and versatile detection of the total electronic spin in open-shell molecular configurations, which can have implications in concepts of molecule-based spin-optical devices. Spin states on individual molecules have been previously measured using electron transport and electron spin-resonance methods^20−26^ but have not been detected optically.

Here, we perform UHV-TERS experiments at an open-shell molecule, which provides an ideal playground for revealing the complex interplay among the charge, spin, and electronic states of a molecule in a nanocavity and its impact on the optical properties. It has been shown that a controlled atomic contact between the STM tip and molecules leads to a dramatic rise of Raman intensity,^27−30^ which is a clear indication of enhancement beyond the electromagnetic field intensification. Therefore, we select a highly tunable single-molecule break-junction system that exhibits a reversible transition between a mixed-valence state and a singly charged radical upon decoupling from a metal substrate.^31−35^ We manipulate a single perylene-3,4,9,10-tetracarboxylic dianhydride (PTCDA) molecule on Ag(111) while simultaneously measuring the electronic spin state and the Raman spectra in the chemical enhancement mode. By correlating the appearance of the characteristic Kondo peak in the differential conductance spectra with the vibrational mode intensities in chemically enhanced TERS, we identify Raman fingerprints of the spin state. A reference TERS mapping of a PTCDA anion on NaCl reveals the resonant character of the spectra, permitting us to perform a complete assignment of the vibrational modes to the observed spectral features based on time-dependent density functional theory (TD-DFT) calculations.

## Results/Discussion

**Figure 1:**
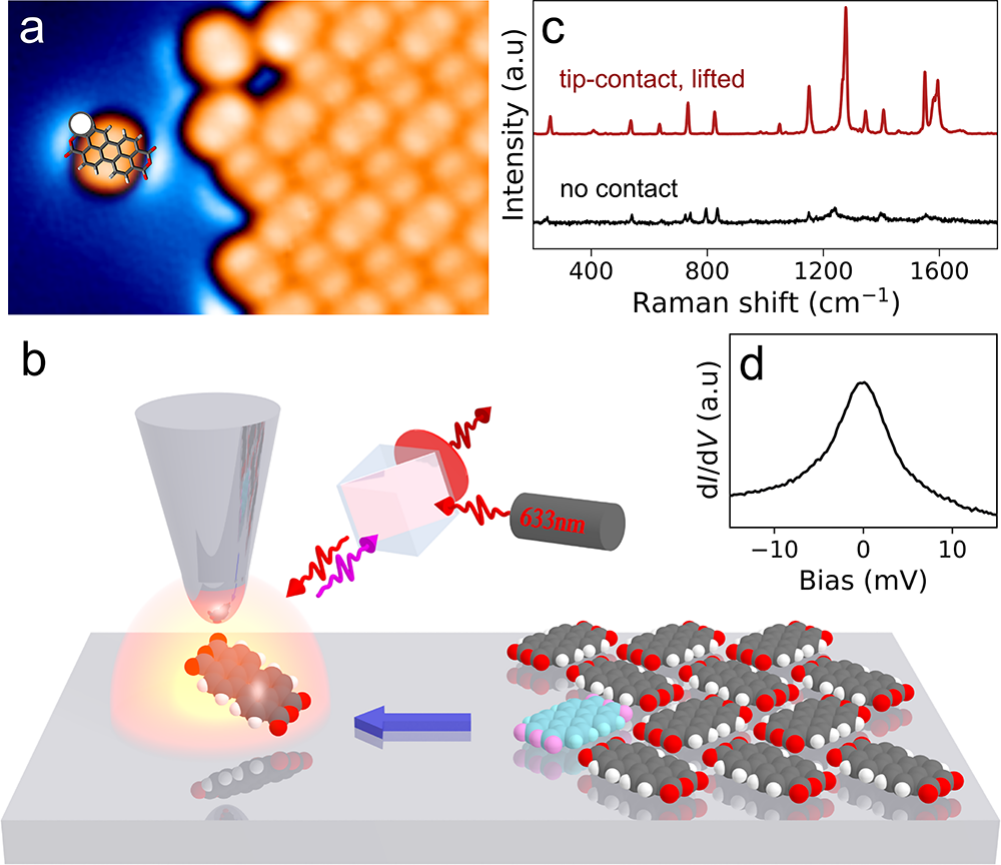
Single-molecule experiment with Raman and spin measurements. (a) Constant-current topographic image of a PTCDA molecule isolated from a 2D assembled layer by deliberate manipulation using the STM probe (−350 mV, 10 pA). (b) Schematic representation of the setup used to contact and decouple the molecule from the substrate (white point in a denotes the point of contact). Subsequently, the enhancement effect of the nanocavity is applied to detect (c) the Raman fingerprint of a single-molecule contacted with the tip (red, 3 s, 1 mV, 1 nA transport current). A spectrum for a molecule on Ag without the tip contact is provided for comparison (black, 50 s, 1 mV, 200 pA tunneling current). (d) d*I*/d*V* spectroscopy around the Fermi level on the contacted and lifted molecule, showing a peak characteristic of the spin 1/2 state.

The setup and methodology of the experiments are schematically shown in Figure 1 and Figure S4. PTCDA molecules are evaporated thermally onto the Ag(111) surface, using the procedure described elsewhere.^33^ On-surface diffusion of the adsorbates promotes the formation of self-assembled islands of molecules. An optically active and atomically sharp Ag-tip is prepared by controlled indentations and voltage pulses, in order to obtain a strong plasmonic response of high intensity spectrally matching the energy of the excitation source (632.8 nm HeNe laser), see Figure S5, and the region of the Raman shift.^36^ Through manipulation, we extract a single PTCDA molecule from the edge of an island and transfer it onto a clean metal area. To this end, we contact the molecule via one of the four carbonyl terminations and drag it laterally away from the island. Subsequently, the molecule is released by retracting the tip several nanometers and inspected by taking an STM image of the area (Figure 1a). After recontacting the molecule, we detach it gradually from the surface by a controlled vertical displacement of the tip while simultaneously measuring Raman spectra using a confocal arrangement (Figure 1b,c). For every step, a differential conductance (d*I*/d*V*) curve is recorded, which is indicative of the electronic spin state of the molecule by showing a prominent Kondo peak centered at the Fermi level when the molecule has a single unpaired electron (Figure 1d).^33^ The molecule bonded to the tip apex can be typically lifted and lowered repeatedly for several cycles in a reproducible manner, as shown in Figures S6 and S7. The Raman signal of a lifted PTCDA is about 2 orders of magnitude more intense compared to the Raman signal obtained in the tunneling regime over the molecule on the surface (Figure 1c).

**Figure 2:**
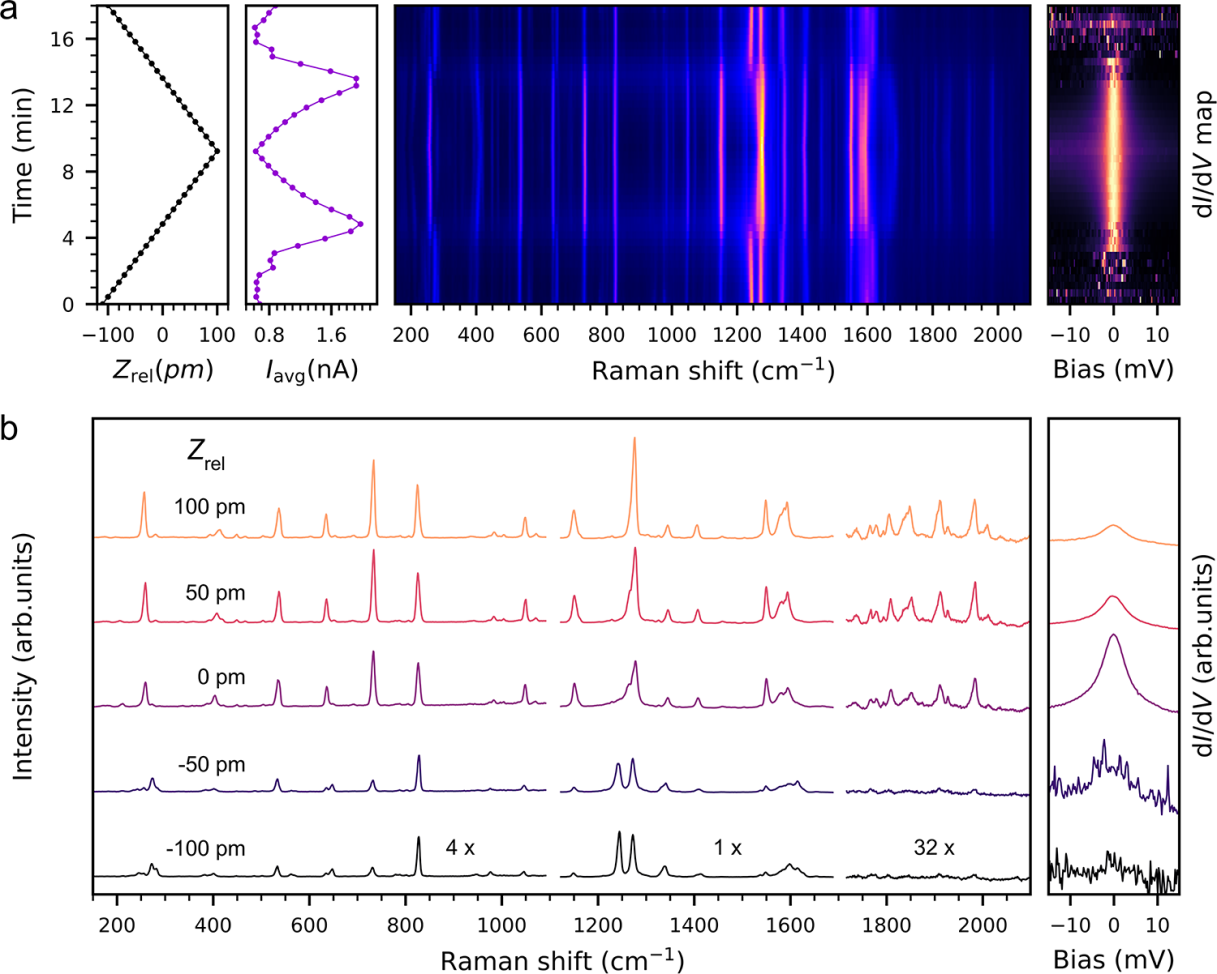
Tracking the spin transition with Raman. (a) Current, TERS and dI/dV intensity (normalized) as a function of the PTCDA height of lifting (*Z*_rel_) from the Ag(111) substrate, relative to the onset of the Kondo signature. The lifting step size was 10 pm. The current measurement is plotted for a bias voltage of 1 mV. (b) Detailed Raman and d*I*/d*V* spectra (non-normalized) at selected heights around the spin transition.

Figure 2a shows a complete cycle of the lifting-laying experiment, with the measured current, TERS, and d*I*/d*V* as a function of the relative height (*Z*_rel_). The as-contacted PTCDA on Ag(111) is still in a mixed valence state, and according to previous analyses its LUMO is situated below the Fermi level of the substrate and nearly doubly occupied.^31,37^ An effective removal of this mixed valence at the lifting height (*Z*_rel_ = 0) is hallmarked by a sharp increase of the conductivity around zero bias voltage. The molecule becomes a single-electron radical (*S* = 1/2), due to the upshift of the LUMO through the Fermi level.^33^ Varying the *Z*_rel_ around this set point from −110 to 100 pm and back (see Figure 2a), we trace the evolution of all the signals simultaneously. The TERS shows reversible transformations coincident with the conductivity maxima, in particular, the pronounced changes of the 1243 cm^–1^ line intensity (see Figure 2b), which strongly correlate with the appearance and disappearance of a strong, clean Kondo signature in the d*I*/d*V* spectra. Such a behavior points out a relation between the changes in the TERS spectrum and the electronic state before and after the spin transition. In addition, with further increasing *Z*_rel_, a decay of the conductivity occurs due to a progressive decoupling of the PTCDA from the substrate, while the TERS overall intensity increases at the same time due to the progressing alignment of the transition dipole with the nanocavity polarization.

**Figure 3:**
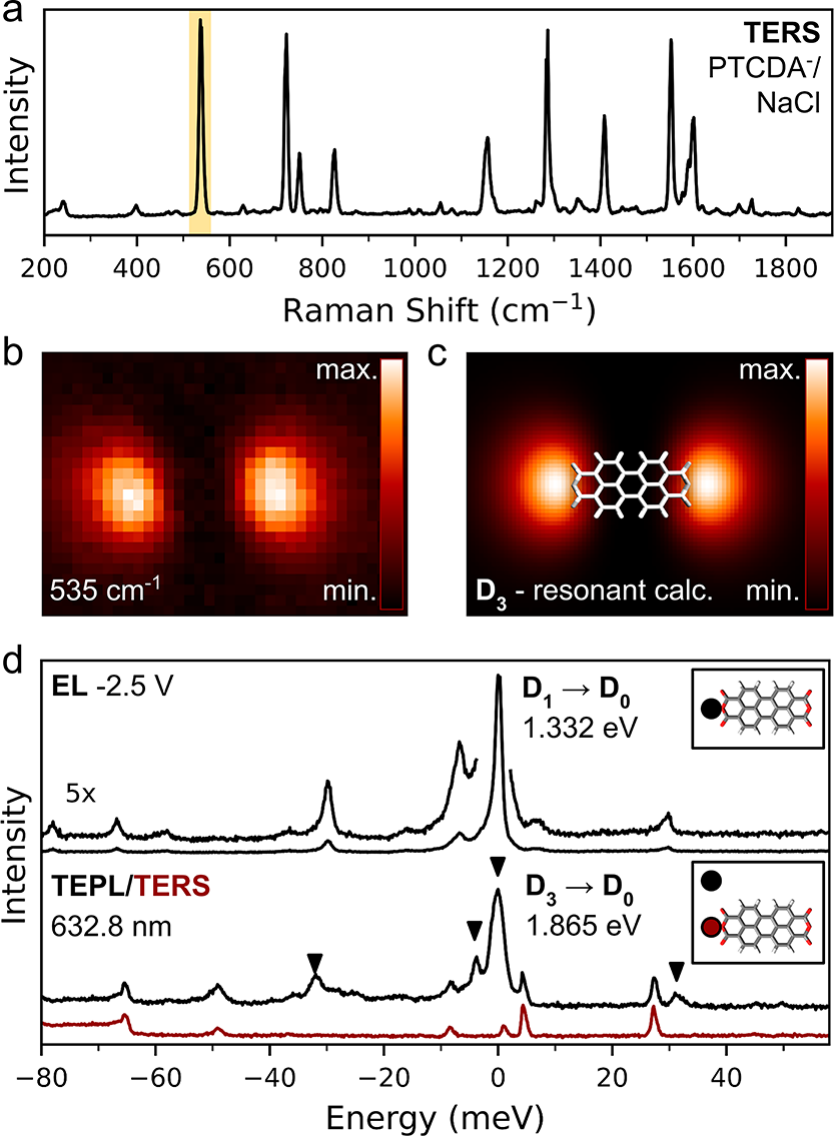
Submolecular mapping of Raman intensity under resonant conditions. (a) Raman spectrum of a single PTCDA^–^on NaCl, acquired at 0.5 V above the oxygen-termination of the PTCDA. (b) Raman intensity map taken from the region around 535 cm^–1^(denoted by yellow band in (a)) in the constant height mode and normalized. Size 3.6 × 2.5 nm^2^, 0.5 V. (c) Simulated Raman map, resonant with the D_3_-state. (d) Comparison of the EL spectrum^10^ corresponding to the D_1_ → D_0_ transition and TEPL spectrum detected using the 632.8 nm excitation, corresponding to the D_3_ → D_0_ transition. The energy scales of the spectra are relative to the energies of the respective transitions. The background Raman spectrum is plotted in red below on the same energy scale for reference (with the energy scale relative to 1.865 eV). The dots in the insets show the approximate locations of the measurements. Black arrowheads denote the peaks attributed to the TEPL contribution. Both TEPL and Raman spectra have the corresponding far-field contribution subtracted.

**Figure 4:**
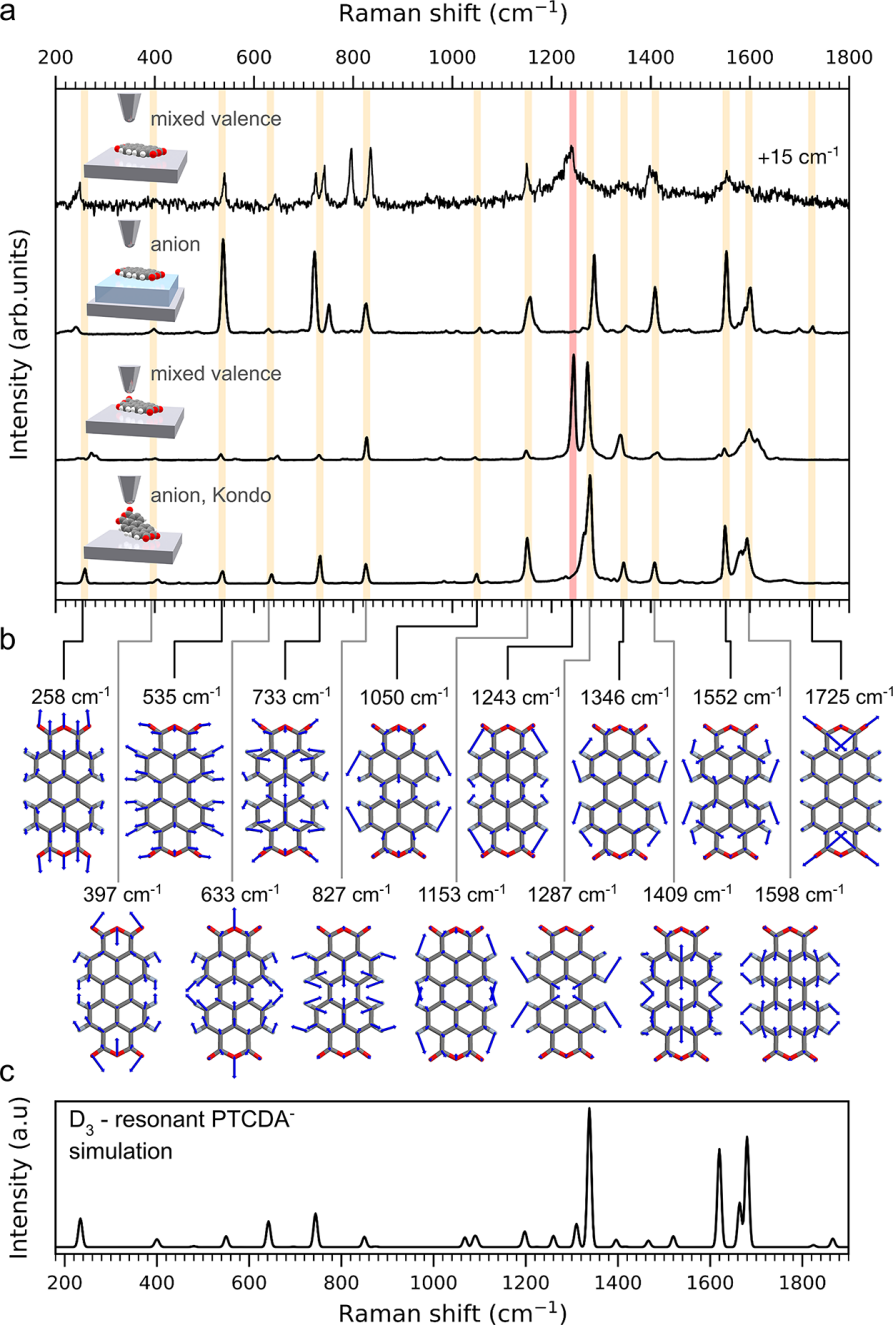
Comparison of observed peaks and assignment to vibrational modes. (a) Top-down comparison of the spectra taken on PTCDA when adsorbed on Ag, on NaCl/Ag, on Ag upon contact with the probe and when lifted. The spectrum of PTCDA adsorbed on Ag is shifted by 15 cm^–1^. The state of the molecule is indicated for each configuration. (b) Dominant A_g_ modes, assigned to the spectral features, based on the agreement between the experimental spectrum in (a) and theoretical simulation in (c). (c) Theoretical Raman spectrum of a PTCDA^–^single radical calculated in resonance with the D_3_ state.

In order to elucidate the regime of the observed intense TERS spectra, to provide grounds for a theoretical simulation, and to support the link of the spectral fingerprints to particular states of the molecule, we performed a reference measurement on the flatly adsorbed PTCDA, decoupled from the Ag substrate by two NaCl layers and without tip contact (Figure 3a). In this configuration, the molecule also adopts a singly charged anionic state (PTCDA^–^), as confirmed by previous works.^10,38^Figure 3d shows that a relatively strong TERS signal is present, similar to the Raman spectrum of the suspended molecule in the *S* = 1/2 state (for comparison see Figure 4a). Although the amplitudes of the two configurations differ, especially in the range below 1000 cm^–1^, likely due to their specific geometries and local environments,^1^ they share a common feature—the diminished peak at 1243 cm^–1^, in contrast to the scenarios where the PTCDA is coupled to the metal substrate and does not have an open-shell character.

The Raman signal is sufficiently strong to take a spatially resolved TERS of the single anion. The intensity map for the prominent line at 535 cm^–1^ (marked in yellow) in Figure 3b, corresponding to the transversal stretching mode (see below), shows a pattern consisting of two lobes localized at both ends of the molecule with the carboxylic terminations. Maps in Figure S8 at the energies of all other dominant peaks manifest a nearly identical characteristic pattern, which resembles the electroluminescence distribution, previously reported for the PTCDA^–^ first excited state transition to the ground state (D_1_ → D_0_).^10^ The strong similarity of the spatial patterns for different vibrational states is in a sharp contrast to the previous spatially resolved TERS measurements, which revealed varying symmetries depending on a particular vibrational state.^1−3,14^ Our observation suggests that in the case of PTCDA^–^, excited by the 632.8 nm light, the TERS scattering is resonant, since when resonantly excited, the Raman intensity is proportional to the Franck–Condon activities of the corresponding vibrations, and its tip-position-dependent amplitude is modulated by the coupling of the nanocavity to the resonant excited state.^39,40^ However, because the energy of the D_1_ → D_0_ transition is 1.33 eV, it cannot contribute resonantly to the Raman scattering at the excitation wavelength 632.8 nm (1.96 eV). The energy of the transition from the second excited state to the ground state (D_2_ → D_0_), being 1.54 eV, is also detuned from the excitation and its transition dipole moment is oriented along the molecular short axis, perpendicular with respect to the observed TERS pattern.^10^ Therefore, we expect a higher excited state with an energy close to the incident light to be responsible for the observed resonant character of the TERS.

In the off-axis position in the peripheral region of the molecule, we are able to detect a prominent spectral feature around 1.865 eV, broader than the rest of the Raman peaks, accompanied by longitudinal stretching mode sidebands at ±31.5 meV, also found in the high-resolution D_1_ → D_0_ electroluminescence spectrum (plotted for comparison in Figure 3d). Based on our TD-DFT calculations, which place the third excited state (D_3_) approximately 290 meV above the D_2_ → D_0_ transition, we attribute the 1.865 eV peak and its sidebands to the D_3_ → D_0_ transition of the PTCDA^–^. This transition has the dipolar moment oriented along the longitudinal axis of the molecule, in accord with the TERS emission pattern in Figure 3b and a very high oscillator strength, stemming from a constructive interference between the dominant electronic transitions involved in the excitation to the many-body D_3_ state, as explained in detail in the Supporting Information. A resonant simulation involving the D_3_ → D_0_ transition density and the point-charge approximation of the nanocavity agrees exceptionally well with the experimental TERS map (shown in Figure 3c). The corresponding simulated D_3_-resonant Raman spectrum of a free-standing PTCDA^–^ (see Figure 4c) is dominated by the highest-symmetry A_g_ modes, in accord with the previously made assessment.^41,42^ The relative Raman activities of the vibrational modes give convincing agreement with the experimental TERS of the suspended PTCDA^–^ in the *S* = 1/2 state, allowing an assignment of the individual vibrational modes and extrapolation to the other measured configurations.

Low-wavenumber modes in the spectra at 258 and 535 cm^–1^ in Figure 4 correspond to the stretching of the molecule in the longitudinal and transversal directions, respectively. The shift in the longitudinal mode frequency during lifting reflects the relaxation of the PTCDA backbone occurring concurrently with the spin transition and the development of the anionic state (detail visible in Figure 2b). The longitudinal mode is also softened in the Ag- and NaCl-adsorbed configurations, probably due to a bond expansion along the corresponding coordinate, stemming from interaction with the substrates. For the molecule on NaCl, the Raman activities of the transversal stretching at 536 cm^–1^ and breathing at 733 cm^–1^ are strongly increased. The latter is also softened to 715 cm^–1^ in both of the adsorbed scenarios and accompanied by an emerging lowered-symmetry mode 33 cm^–1^ higher in energy. We show in the Supporting Information that the rise in activities of the high-symmetry modes can be adequately described solely by the Franck–Condon principle, without the need to include Herzberg–Teller contributions. We can therefore infer that upon transition to D_3_, in comparison to the other configurations, the molecule on NaCl undergoes a larger distortion along these particular modes.

The most prominent spectral lines at 1243, 1287, and 1552 cm^–1^, are all vibrational modes with a significant C–H bending character. The line at 1243 cm^–1^ (denoted with a red bar in Figure 4a) is the only visible peak that is significantly reduced (and shifted to higher frequency) during the development of the single-anionic state and can be considered a hallmark of the PTCDA spin transition. Conversely, the 1287 cm^–1^ line is present in all situations where the spectra are chemically enhanced due to contact with the tip or resonant, *i.e*., always except for the molecule on Ag. Other less intense vibrational peaks are common in all of the probed cases at 1153 and 1346 cm^–1^ and can be attributed to the C–H scissoring and bending modes. Higher-order longitudinal stretching peaks appear at 1409 and 1598 cm^–1^. Interestingly, the C–O stretching found at 1725 cm^–1^ for the unperturbed PTCDA^–^ on NaCl, is shifted about 60 cm^–1^ lower and significantly broadened for the lifted molecule. This is an indication of the C–O bond weakening due to the chemical interaction of the oxygen atoms with the tip and the metal substrate.

## Conclusions

In summary, we have studied the TERS spectrum of single PTCDA molecule in four basic configurations of various electronic and adsorption states: (i) adsorbed on metal in a mixed valence state, (ii) adsorbed on a decoupling NaCl bilayer in open-shell *S* = 1/2 state, (iii) adsorbed on metal and contacted by the tip still in the mixed valence state, and (iv) suspended between the Ag(111) and the tip with the *S* = 1/2. We have found that the TERS signals of the PTCDA measured with a conventional 632.8 nm laser excitation are significantly increased by physical contact with the tip and by the Raman scattering resonant with the D_0_ ↔ D_3_, which was found by TEPL. Comparison of the experimental TERS of the *S* = 1/2 state of the singly charged PTCDA anion with the resonant-Raman TD-DFT calculations yields a good agreement, allowing an unambiguous identification of the Raman-active modes. By following the evolution of the Raman activities of individual modes in the spectra during lifting/laying of the molecule from/to the metal substrate and comparing them to PTCDA^–^ adsorbed on NaCl, we have concluded that the appearance of Kondo resonance (a clear hallmark of the *S* = 1/2 state) anticorrelates with the high intensity of a C–H bending mode at 1243 cm^–1^. Therefore, this particular mode can be used as an indicator of the spin state. With further lifting of the PTCDA^–^ from the metal substrate, the overall TERS intensity is increasing due to the progressing alignment of the transition dipole with the nanocavity polarization. The results of this work bring the prospects of measurements when a higher excited state resonance, tip enhancement, or tip-contact chemical enhancement effect will work in combination and result in genuinely high Raman yields on single molecules, while preserving the intrinsic Raman activities of individual vibrational modes. We envisage the exciting possibility that characteristic changes in single-molecule Raman spectra as reported here could be engineered and employed in organic-based spin-optical transducers.

## Methods/Experimental Section

The experiments were performed in a LT-STM (Createc GmbH) operating at 7K and in an ultrahigh vacuum (UHV) environment below 5 × 10^–11^ mbar base pressure. The Ag(111) single crystal was prepared by standard cycles of Ar^+^ sputtering and annealing at 550 °C. To obtain islands with two to four layers of NaCl, it was thermally evaporated at 610 °C on the surface held at 120 °C for 3–4 min. PTCDA molecules were sublimated at 350 °C onto both Ag(111) and NaCl/Ag(111) samples, kept at room temperature or at 5 K, respectively. The optical setup was based on a confocal arrangement. d*I*/d*V* curves were measured with a lock-in technique using modulation amplitude 2 mV. Further details of the experimental methods are described in the Supporting Information.

We modeled the spectral response and photon map using time-dependent density functional theory (TDDFT) as implemented in Gaussian 16.^43^ The spectral response was modeled using the resonance Raman approach considering both the Herzberg–Teller and Franck–Condon activities of the vibrations^44^ (further details are in the Supporting Information, where we discuss the resonance Raman spectra calculated for the S_0_ ↔ S_1_, D_0_ ↔ D_1_, and D_0_ ↔ D_3_ transitions). The photon map of vibronic peaks was calculated by assuming that a single plasmon mode of the tip couples to the molecular D_0_ ↔ D_3_ transition. Details of the implementation are also discussed in the Supporting Information.

## Supporting Information Available

Time-dependent density-functional-theory calculations, calculation of the Raman map, optical setup, and nanocavity plasmon tuning, reproducibility of the lifting data, and Raman maps at different vibrational modes (PDF)
