# Supplementary material for: Resonant Tip-Enhanced Raman Spectroscopy of a Single-Molecule Kondo System
Source: ACS Nano. 2024 May 7;18(20):13164–70. doi: 10.1021/acsnano.4c02105 (PMC11112976; doi:10.1021/acsnano.4c02105)
Supplement: Supplementary file 1 — nn4c02105_si_001.pdf [file nn4c02105_si_001.pdf]

Supporting Information for

Resonant Tip-Enhanced Raman Spectroscopy of a Single-Molecule Kondo System

Rodrigo Cezar de Campos Ferreira[1], Amandeep Sagwal[1,2], Jiří Doležal[1,3], Sofia Canola[1], Pablo Merino[4], Tomáš Neuman[1], Martin Švec[1,5]*

[1] Institute of Physics, Czech Academy of Sciences; Cukrovarnická 10/112, CZ16200 Praha 6, Czech Republic

[2] Faculty of Mathematics and Physics, Charles University; Ke Karlovu 3, CZ12116 Praha 2, Czech Republic

[3] Institute of Physics, École Polytechnique Fédérale de Lausanne, CH-1015 Lausanne, Switzerland

[4] Instituto de Ciencia de Materiales de Madrid; CSIC, Sor Juana Inés de la Cruz 3, E28049 Madrid, Spain

[5] Institute of Organic Chemistry and Biochemistry, Czech Academy of Sciences; Flemingovo náměstí 542/2. CZ16000 Praha 6, Czech Republic

Theoretical calculations

Time-dependent density-functional-theory calculations

To theoretically describe the resonance Raman spectra we perform time-dependent density-functional theory (TDDFT) modeling of the optical response of the perylene-3,4,9,10-tetracarboxylic dianhydride (PTCDA) molecule. Our approach neither does take into account geometrical deformations of the molecule due to its attachment to the tip and the substrate nor it considers the electronic coupling of the molecule with the metallic electrodes, as the description of such phenomena is beyond the scope of the present work. We therefore resort to calculation of the molecule optical response in a vacuum, considering that upon lifting, the electronic structure of the molecule corresponds to the one of its fully decoupled counterpart. In particular we perform TDDFT calculations using Gaussian 16 revision A.03 and C.01[1] using the double zeta Gaussian basis set including polarization functions 6-31G* and the range-separated hybrid functional ωB97XD.[2]

We first optimize the molecular geometry in the ground state and calculate the molecular vibrations considering that the ground state is (i) a neutral singlet (S_0) and (ii) a negative doublet (D_0). We perform a geometry optimization and vibrational analysis in selected excited states of the molecule. For excited state S_1 of the neutral molecule and states D_1, D_3 of the negative molecule, we calculate resonance Raman response using the Franck-Condon and Herzberg-Teller (FCHT) analysis implemented in Gaussian 16. In particular, since the molecule has been shown to be singly negative when decoupled by lifting from a metal substrate,[3,4] we compare the experimentally observed spectra to the electronic transitions between the ground state and the first and third excited state (D_1^- and D_3^-) which are the two lowest lying states featuring a transition dipole moment aligned along

the PTCDA long axis. We also calculate the Raman spectrum in the first excited (S_1) state of the neutral molecule.

We summarize the results of the calculations of the electronic structure in Fig.S1. Figure S1a shows a schematic diagram of the many-body ground and the first excited singlet state of the neutral molecule. The energy of the transition evaluated in the ground-state geometry is shown in the diagram (or in the excited state geometry in the brackets). The corresponding calculated transition density of the $S_0 \leftrightarrow S_1$ transition is shown. The transition density shows a dipolar moment along the molecule's long axis. Alongside with the many-body states we show their corresponding orbital occupations in the configuration diagram in the inset. The ground-state configuration and orbitals are derived from the underlying DFT calculation. For the excited state, we derive the configuration from the dominant electron-hole pair transitions contributing to the excitation as calculated by the linear-response TDDFT. The orbitals (highest-occupied - HOMO, lowest unoccupied - LUMO, LUMO+1 and LUMO+2) occupations corresponding to the S_0 state are shown on the right for completeness. From the diagrams we see that the HOMO-LUMO transition dominantly contributes to $S_0 \leftrightarrow S_1$.

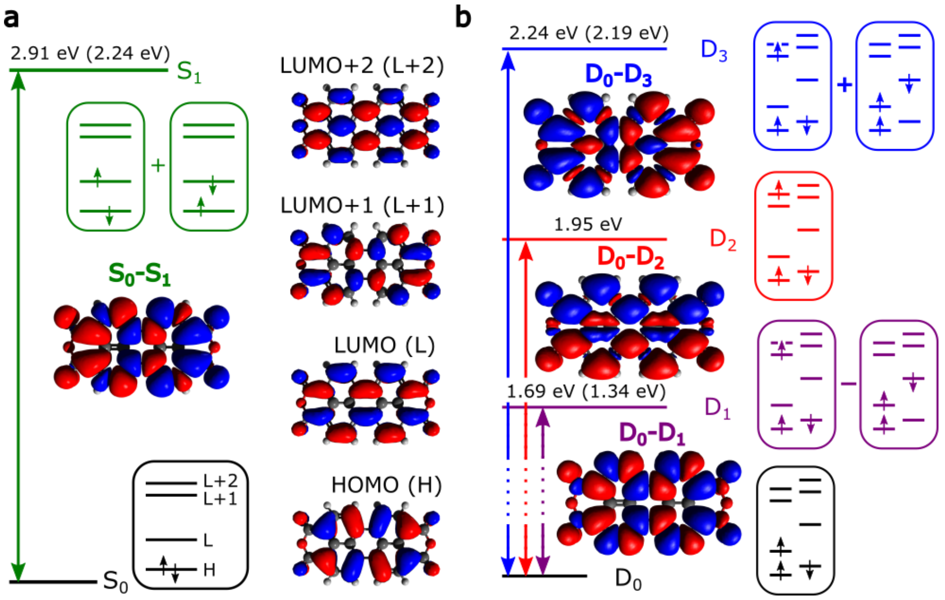

Fig.S1: *Electronic structure of the neutral and negative PTCDA molecule. (a) The transition density of the $S_0 \leftrightarrow S_1$ transition is shown alongside the diagram showing the excitation energy and the schematic electron configuration diagram of the electronic states. The molecular orbitals (HOMO - H, LUMO - L, LUMO+1 - L+1, and LUMO+2 - L+2) obtained for the neutral molecule are shown next to the many-body diagram. (b) Excitations of the negative molecule with the corresponding transition densities, excitation energies, and electron configuration diagrams. The energy labels in the diagram mark the energies calculated as vertical transitions in the ground-state (and in the excited-state in the brackets) geometry.*

Figure S1b displays the electronic structure of the negative molecule. The first three excited states are shown with the corresponding transition energies in the ground-state (excited-state) geometry labeled in the diagram. The three corresponding transition

densities ($D_0 \leftrightarrow D_1$ - purple label, $D_0 \leftrightarrow D_2$ - red label, $D_0 \leftrightarrow D_3$ - blue label) are shown. The transition densities corresponding to $D_0 \leftrightarrow D_1$ and $D_0 \leftrightarrow D_3$ feature a dipole moment oriented along the long axis of the molecule, in contrast to $D_0 \leftrightarrow D_2$ that has a dipole moment along the short axis of the molecule. We also show the configuration diagram showing the orbital occupations in the doublet states. In the open-shell configuration we distinguish the orbitals for the spin up and spin down electrons as the underlying DFT calculation uses the spin-unrestricted ansatz and thus the orbitals are calculated independently for the two spin channels. Despite this, the orbitals can still be associated with the ones of the neutral molecule shown in Fig.S1a. The excited-state configurations are again derived from the dominant electron-hole pair transitions contributing to the respective excitations. The configuration diagrams are color-coded using the same labeling scheme as for the transition densities. From the configurations it is apparent that the $D_0 \leftrightarrow D_1$ and $D_0 \leftrightarrow D_3$ transitions contain the same electron-hole pairs, but the electron-hole pair superposition differs in sign. As a consequence, the transition dipole moment of the $D_0 \leftrightarrow D_3$ is significantly larger than that of the $D_0 \leftrightarrow D_1$ transition. This is because in the first case the contributing electron-hole pairs superpose constructively, whereas in the latter case destructively. The $D_0 \leftrightarrow D_3$ transition can thus be expected to significantly contribute to the optical response of the molecule, including the (near) resonance-Raman response at the energy of the laser used in the experiments.

We now focus on the calculation of resonance Raman spectra corresponding to laser tuned close to the $D_0 \leftrightarrow D_3$ transition. Since in the experiment the incident laser may not be exactly resonant with the electronic transition, we test the effect of the incident-laser detuning and calculate the resonance Raman spectra for several frequencies of the incident laser. The results normalized to the maximum are shown in Fig.S2a in the form of a waterfall plot. The spectral peaks vary their relative intensities as the laser frequency is tuned and we observe that particularly the peaks of frequency <1000 cm^{-1} have relatively larger intensity in the range of incident laser frequencies between approximately 16000-19000 cm^{-1} and their relative weight in the spectrum peaks around 17800 cm^{-1}. We also see that the peaks between 1600 cm^{-1} and 1700 cm^{-1} vary their relative intensity when the laser frequency is detuned. We note that the spectrum at 17000 cm^{-1} is used in the main text as it is the best match with the experiment.

In Fig.S2b-d we compare the resonance Raman spectra calculated for the three electronic transitions: (b) $D_0 \leftrightarrow D_1$, (c) $D_0 \leftrightarrow D_3$, and (d) $S_0 \leftrightarrow S_1$. We observe that the spectra for the respective transitions differ considerably. The Raman peaks of the lower-energy vibrations are more pronounced in the $D_0 \leftrightarrow D_3$ spectrum than in the $D_0 \leftrightarrow D_1$ and spectra $S_0 \leftrightarrow S_1$. This finding supports the interpretation of the experimental spectrum as a resonance Raman spectrum of the $D_0 \leftrightarrow D_3$ transition.

In Fig.S3 we show the resonance Raman spectra of the three transitions: (a) $D_0 \leftrightarrow D_1$, (b) $D_0 \leftrightarrow D_3$, and (c) $S_0 \leftrightarrow S_1$. They have been calculated using the full Franck-Condon-Herzberg-Teller formalism (black lines), and the spectra calculated only using the Franck-Condon activity of the vibrations (red lines). We see that the inclusion of the Herzberg-Teller formalism does not lead to any significant differences of the spectra with

respect to the purely Franck-Condon spectra, so we can conclude that the Franck-Condon formalism alone is sufficient to obtain a good theoretical description of this system.

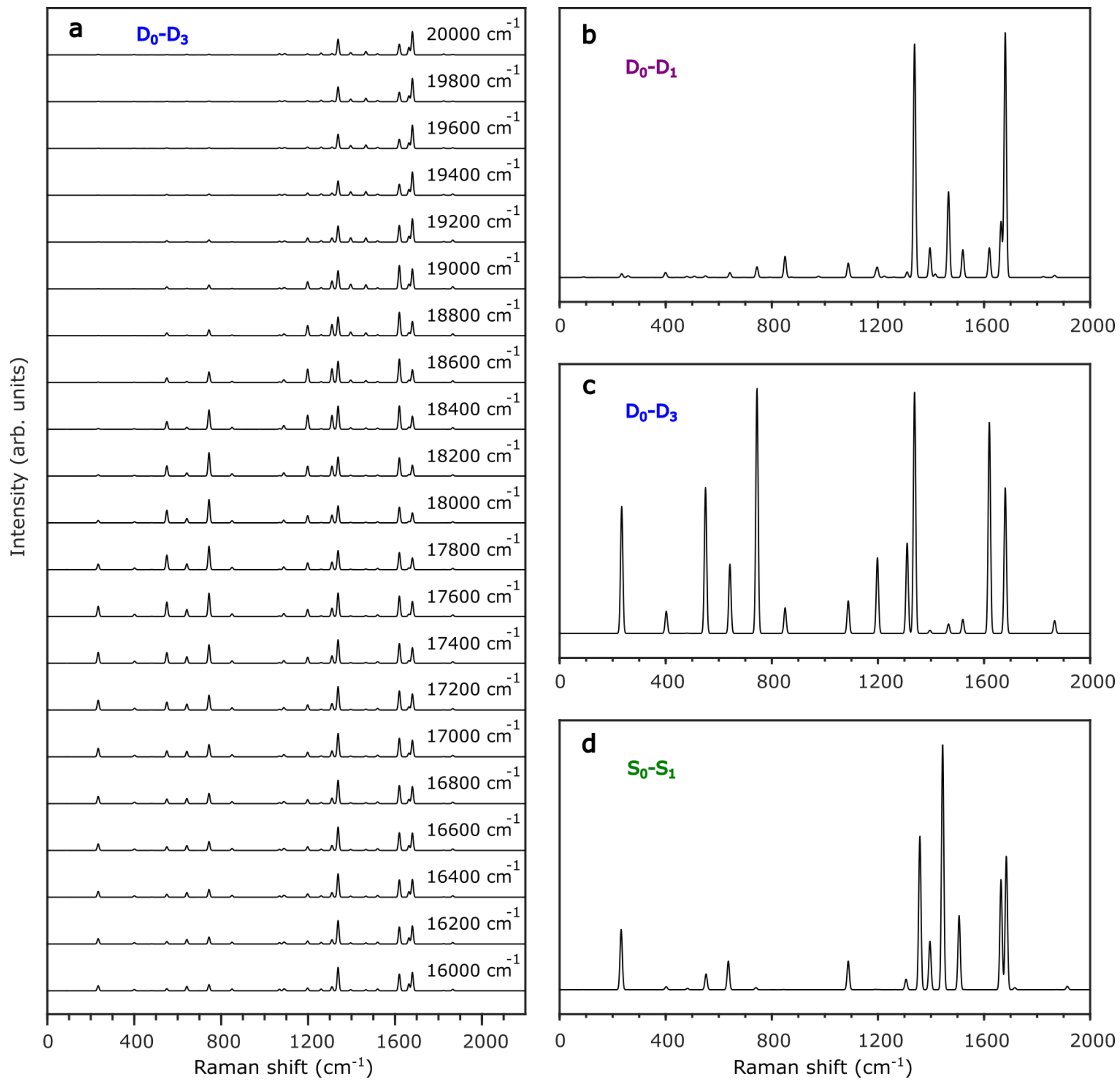

Fig.S2: *Near-resonance Raman spectra of PTCDA corresponding to different electronic transitions. (a) Spectra corresponding to Raman spectra of the* $D_0 \leftrightarrow D_3$ *transition assuming different excitation frequencies (the 0-0 transition being at 17452.56 cm*$^{-1}$*). (b)-(d) Resonance Raman spectra assuming exact tuning of the excitation laser with the (b)* $D_0 \leftrightarrow D_1$ *transition, (c)* $D_0 \leftrightarrow D_3$ *transition, and (d)* $S_0 \leftrightarrow S_1$ *transition.*

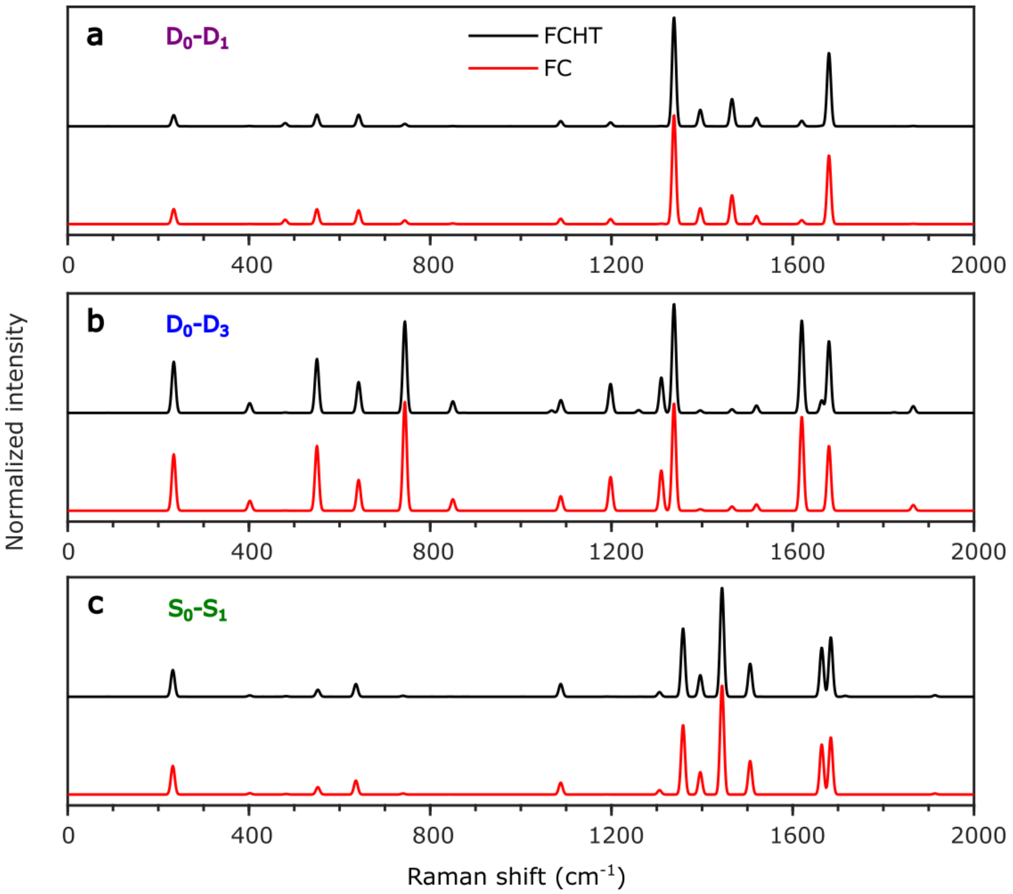

Fig.S3: *Comparison of Raman spectra calculated using the full Franck-Condon-Herzberg-Teller approach (FCHT - black lines) and spectra calculated using only the Franck-Condon principle (FC - red lines). The spectra were calculated assuming the exact tuning of the laser to the transition energy of the (a)* $D_0 \leftrightarrow D_1$ *transition, (b)* $D_0 \leftrightarrow D_3$ *transition, and (c)* $S_0 \leftrightarrow S_1$ *transition.*

Calculation of the Raman map

In Fig.3c of the main text we show the calculated Raman photon intensity map (Raman map) recorded as a function of the lateral position of the tip. We calculate the Raman map assuming that the $D_0 \rightarrow D_3$ transition is close to the resonance with the excitation energy. In this situation the Raman signal recorded as a function of the position of the tip r_{tip} can be approximated as $I_R(r_{tip}) \propto |g_{pl}(\omega_L, r_{tip})|^2 |g_{pl}(\omega_{St}, r_{tip})|^2 I_0$, where I_0 is the intensity of the incident laser, and $g_{pl}(\omega, r_{tip})$ is the plasmon-exciton coupling that we calculate as $g_{pl}(\omega, r_{tip}) \propto \int \phi_{pl}(r - r_{tip}; \omega)\rho_{D_0 \rightarrow D_3}(r)d^3r$, with $\phi_{pl}(r - r_{tip})$ being an approximation of the quasi-static electric potential of the plasmon mode (representing its mode profile), and $\rho_{D_0 \rightarrow D_3}(r)$ being the transition density of the $D_0 \rightarrow D_3$ transition. We would like to point out that in the expression for $I_R(r_{tip})$, one commonly finds the plasmon-enhancement factors f_{pl} (defined as $f_{pl} = |E_{loc}|/|E_{inc}|$ with E_{inc} and E_{loc} being the incident and local electric field, respectively) instead of the plasmon-exciton coupling. The use of the factor f_{pl} is justified

when the molecular response can be treated in the dipolar approximation. In the case of plasmon-exciton coupling in a STM and considering near-resonant Raman,[5,6] it is the coupling of the transition density of a particular excitonic transition with a particular localized plasmonic mode treated beyond the dipole approximation that determines the scattering properties of the molecule (assuming that the Franck-Condon mechanism is at play). The plasmon-exciton coupling should also be generally evaluated at the frequency of the incident light ω_L and at the frequency of the Stokes photon ω_{St}. However, with the assumption that the plasmonic mode profile does not significantly vary with frequency (i.e. that a single plasmon mode is being excited and the same mode participates in the emission process) we obtain the simplified expression $I_R(r_{tip}) \propto |g_{pl}(r_{tip})|^4$ that we use to calculate the map in Fig.3c. We approximate the plasmonic mode profile by the potential of a pair of point charges of equal magnitude and opposite sign positioned 1.5 nm above and 2.5 nm below the plane of the molecule, respectively, at the same lateral position. This potential sufficiently approximates the electric field of a localized gap plasmon in the gap; we note that the details of the field distribution (beyond the field localization) do not play a significant role in the resulting shape of the photon map.

Experimental details

Optical setup and nanocavity plasmon tuning

In the optical setup used for the experiments, schematically depicted in Fig.S4, the excitation source was a He-Ne (632.8 nm) continuous-wave laser, collimated with an *f* = 15 mm lens, guided through a ND filter, half-wave plate, polarizer and a noise eater, to form a stable-intensity beam with polarization along the tip-sample axis. Typical total beam power used for the experiments varied from 50-200 uW. The beam was focused into the scanning probe microscope (SPM) junction by another internal SPM lens (also with *f* = 15 mm). The outgoing light from the SPM nanocavity was filtered using a 633 nm bandpass edge filter. The tip-enhanced Raman spectroscopy (TERS) and tip-enhanced photoluminescence (TEPL) spectra were measured in cumulative mode by an Andor Kymera 328i spectrograph with a 1200 grooves/mm, 500 nm blaze grating, connected to a custom control computer and homemade control software based on the Andor software development kit. The Ag tips made of 25 µm diameter Ag wire were sharpened by focused Xe^+ ion beam. Further cleaning by head-on Ar^+ sputtering was done before insertion into the SPM head and final shaping was done using nanoindentations and voltage pulses.

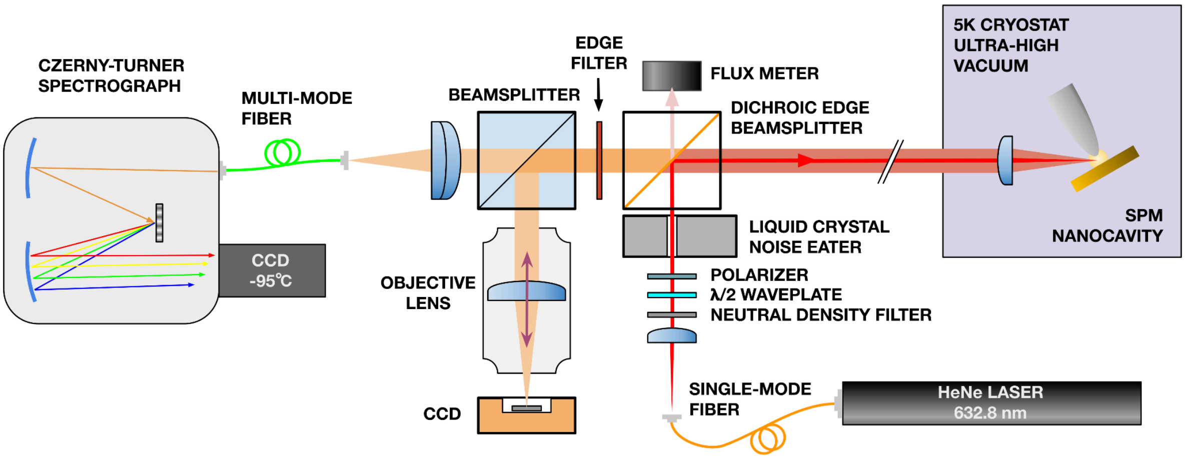

Fig.S4: *The scheme of the confocal optical setup for the measurements of single-molecule TERS, TEPL and EL at 5K and cryogenic conditions.*

The nanocavity frequency spectrum was determined by electroluminescence and was tuned to cover the range of the excitation energy and the Raman scattering. Test of the photon coupling to the nanocavity was performed using the field emission resonance measurements with a lock-in technique. As the efficient coupling causes electron energy conversion, it manifests itself as a downshift of the electron tunneling resonances, corresponding to the excitation source energy (see Fig.S5)[7].

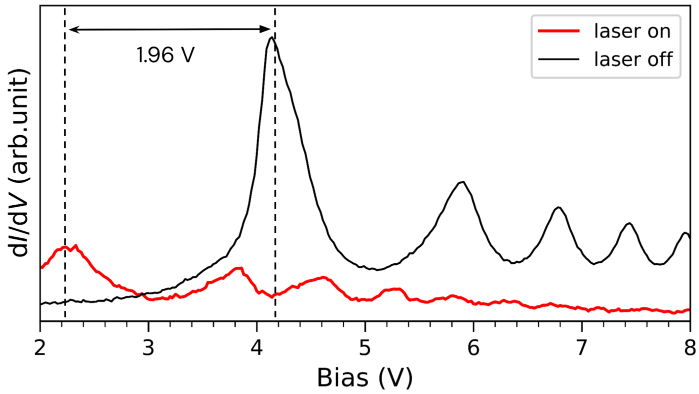

Fig.S5: *Field-emission resonances, measured as dI/dV at constant current feedback with an Ag tip on a clear Ag(111) surface as a function of the irradiation being on/off. The current setpoint was 100 pA, lock-in modulation amplitude 50 mV.*

Reproducibility of the lifting data

We performed several sessions of the lifting experiments, with various tips and PTCDA molecules. We have typically achieved reproducibility of the measured dI/dV and Raman spectra over more than two complete cycles. Figs. S6 and S7 show additional two datasets, measured with the 600/nm grating (at lower resolution with respect to the data presented in Fig.2 which was measured with the 1200/nm grating for high-resolution).

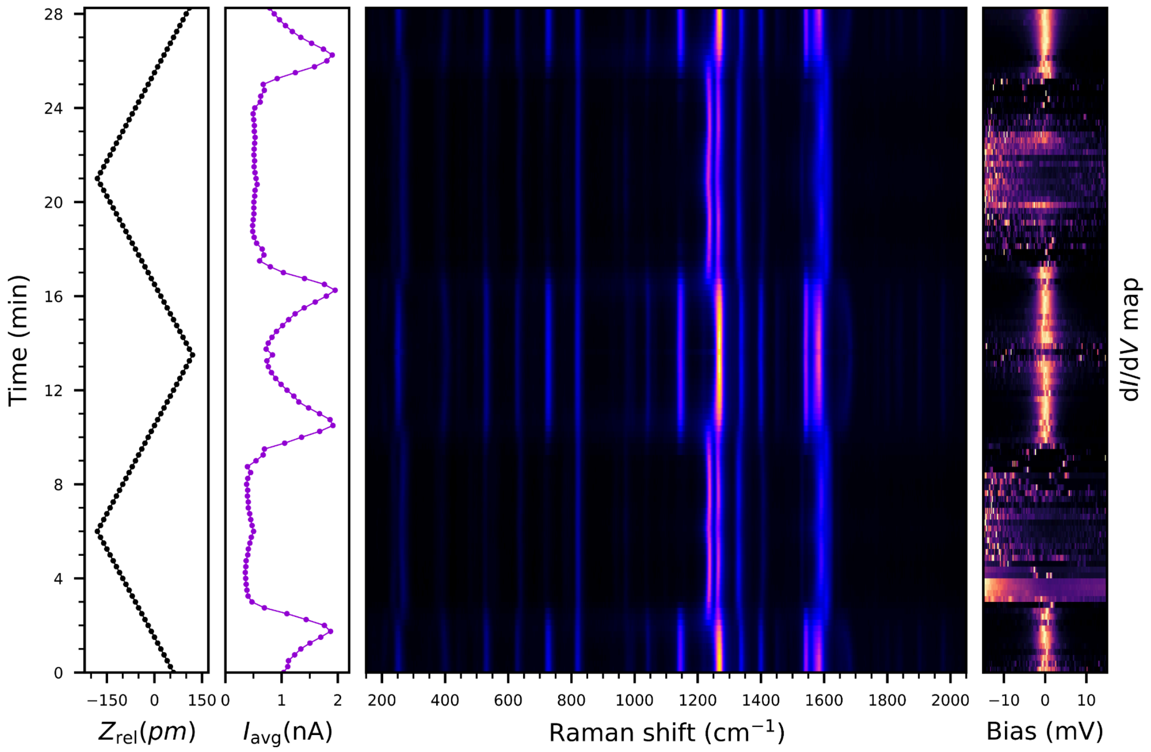

Fig.S6: *Current (I_{avg}), TERS and normalized dI/dV intensity (from left to right) as a function of the PTCDA height of lifting (Z_{rel}) from the Ag substrate, relative to the onset of the Kondo signature. The lifting step size was 10 pm.*

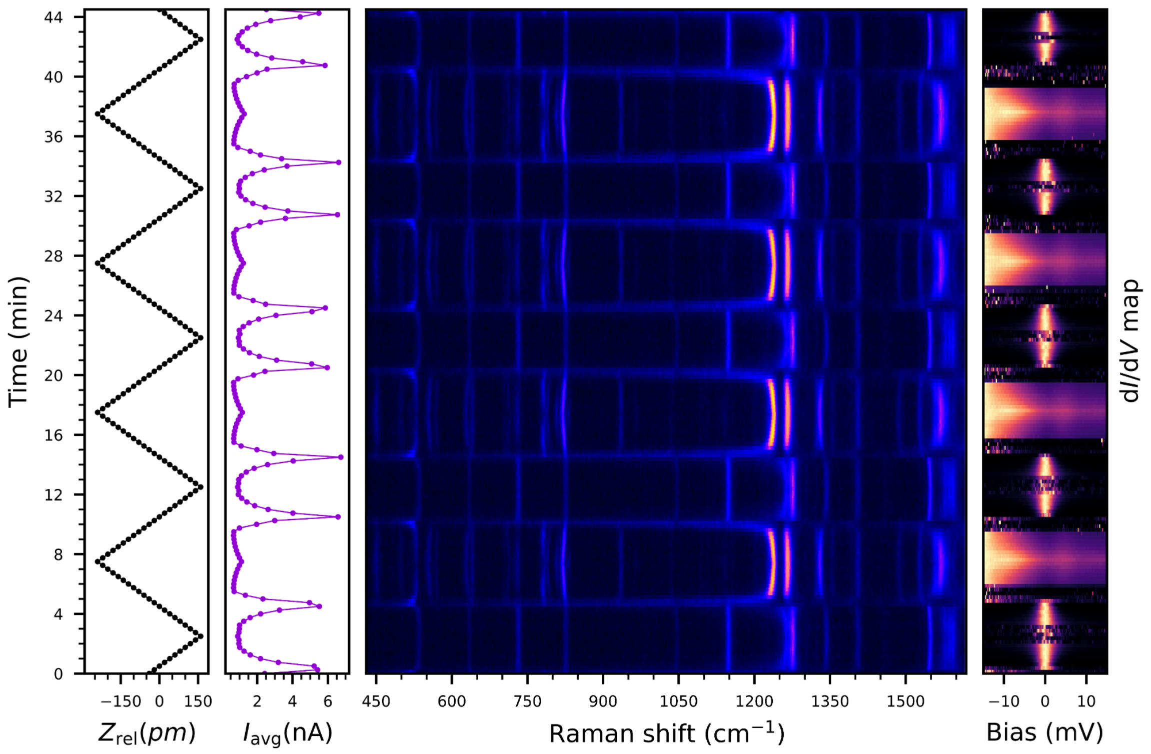

Fig.S7: *Current (I_{avg}), TERS and normalized dI/dV intensity (from left to right) as a function of the PTCDA height of lifting (Z_{rel}) from the Ag substrate, relative to the onset of the Kondo signature. The lifting step size was 20 pm.*

Raman maps at different vibrational modes

The Raman spectroscopy was measured as a function of the lateral position of the tip above the PTCDA⁻ on two monolayers of NaCl. In addition to the map shown in Fig.3b, maps for other vibrational peaks and the background are presented in Fig.S8. The overall distributions of the intensity in the maps for individual modes are very similar, strongly indicating that the scattering process is resonant with the $D_0 \leftrightarrow D_3$ transition density. This interaction is strongest at the extremities of the molecule, reflecting that the scattering properties of the molecule are primarily driven by the coupling of excitonic transition with the localized plasmonic mode of the nanocavity (as explained above in the theoretical explanation of the map simulation).

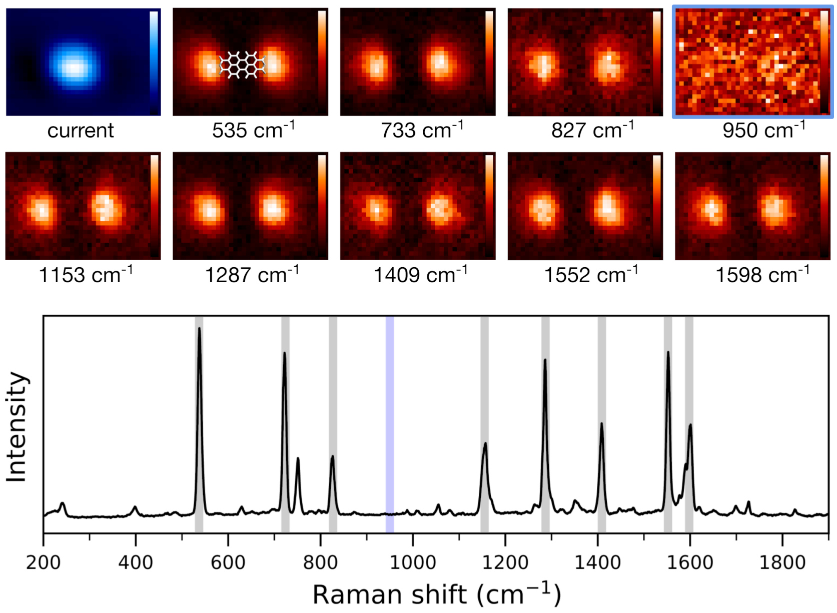

Fig.S8: *The constant-height current and TERS maps on the PTCDA on two layers of NaCl on Ag(111) (top), plotted for all dominant intensity peaks in the overall TERS spectrum (bottom). The size of all maps is 36 x 25 pixels, 3.6 x 2.5 nm^2. Each map is normalized and the intensity is color-mapped using the same color-intensity scale in which the darkest tone corresponds to the minimum and the brightest to the maximum intensity value in a map. The spectra were measured at 0.5 V. The color scale (blue-white) of the measured current corresponds to 0-7 pA.*

Supporting Information References:

(1) Frisch, M. J.; Trucks, G. W.; Schlegel, H. B.; Scuseria, G. E.; Robb, M. A.; Cheeseman, J. R.; Scalmani, G.; Barone, V.; Petersson, G. A.; Nakatsuji, H.; Li, X.; Caricato, M.; Marenich, A. V.; Bloino, J.; Janesko, B. G.; Gomperts, R.; Mennucci, B.; Hratchian, H. P.; Ortiz, J. V.; Izmaylov, A. F.; Sonnenberg, J. L.; Williams-Young, D.; Ding, F.; Lipparini, F.; Egidi, F.; Goings, J.; Peng, B.; Petrone, A.; Henderson, T.; Ranasinghe, D.; Zakrzewski, V. G.; Gao, J.; Rega, N.; Zheng, G.; Liang, W.; Hada, M.; Ehara, M.; Toyota, K.; Fukuda, R.; Hasegawa, J.; Ishida, M.; Nakajima, T.; Honda, Y.; Kitao, O.; Nakai, H.; Vreven, T.; Throssell, K.; Montgomery, J. A., J.; Peralta, J. E.; Ogliaro, F.; Bearpark, M. J.; Heyd, J. J.; Brothers, E.

N.; Kudin, K. N.; Staroverov, V. N.; Keith, T. A.; Kobayashi, R.; Normand, J.; Raghavachari, K.; Rendell, A. P.; Burant, J. C.; Iyengar, S. S.; Tomasi, J.; Cossi, M.; Millam, J. M.; Klene, M.; Adamo, C.; Cammi, R.; Ochterski, J. W.; Martin, R. L.; Morokuma, K.; Farkas, O.; Foresman, J. B.; Fox, D. J. Gaussian 16, Revision A.03; Gaussian, Inc., Wallingford CT. 2016.

(2) Chai, J. Da; Head-Gordon, M. Long-Range Corrected Hybrid Density Functionals with Damped Atom–Atom Dispersion Corrections. *Phys. Chem. Chem. Phys.* **2008**, *10*, 6615–6620.

(3) Temirov, R.; Lassise, A.; Anders, F. B.; Tautz, F. S. Kondo Effect by Controlled Cleavage of a Single-Molecule Contact. *Nanotechnology* **2008**, *19*, 065401.

(4) Žonda, M.; Stetsovych, O.; Korytár, R.; Ternes, M.; Temirov, R.; Raccanelli, A.; Tautz, F. S.; Jelínek, P.; Novotný, T.; Švec, M. Resolving Ambiguity of the Kondo Temperature Determination in Mechanically Tunable Single-Molecule Kondo Systems. *J. Phys. Chem. Lett.* **2021**, *12*, 6320–6325.

(5) Neuman, T.; Esteban, R.; Giedke, G.; Schmidt, M. K.; Aizpurua, J. Quantum Description of Surface-Enhanced Resonant Raman Scattering within a Hybrid-Optomechanical Model. *Phys. Rev. A* **2019**, *100*, 043422.

(6) Neuman, T.; Esteban, R.; Casanova, D.; García-Vidal, F. J.; Aizpurua, J. Coupling of Molecular Emitters and Plasmonic Cavities beyond the Point-Dipole Approximation. *Nano Lett.* **2018**, *18*, 2358–2364.
